# Evidence for Involvement of *GNB1L* in Autism

**DOI:** 10.1002/ajmg.b.32002

**Published:** 2011-11-16

**Authors:** Ying-Zhang Chen, Mark Matsushita, Santhosh Girirajan, Mark Lisowski, Elizabeth Sun, Youngmee Sul, Raphael Bernier, Annette Estes, Geraldine Dawson, Nancy Minshew, Gerard D Shellenberg, Evan E Eichler, Mark J Rieder, Deborah A Nickerson, Debby W Tsuang, Ming T Tsuang, Ellen M Wijsman, Wendy H Raskind, Zoran Brkanac

**Affiliations:** 1 Department of Medicine (Medical Genetics), University of WashingtonSeattle, Washington; 2 Department of Genome Sciences, University of WashingtonSeattle, Washington; 3 Department of Psychiatry and Behavioral Sciences, University of WashingtonSeattle, Washington; 4 Department of Speech and Hearing Sciences, University of WashingtonSeattle, Washington; 5 Department of Psychiatry, University of North Carolina Chapel HillChapel Hill, North Carolina; 6 Department of Psychiatry and Neurology, University of PittsburghPittsburgh, Pennsylvania; 7 Department of Pathology and Laboratory Medicine, University of Pennsylvania School of MedicinePhiladelphia, Pennsylvania; 8 Howard Hughes Medical InstituteSeattle, Washington; 9 VISN-20 Mental Illness Research, Education, and Clinical Center, Department of Veteran AffairsSeattle, Washington; 10 Department of Psychiatry University of CaliforniaSan Diego, La Jolla, California; 11 Department of Biostatistics, University of WashingtonSeattle, Washington

**Keywords:** 22q11.2, translocation, neurodevelopmental disorders

## Abstract

Structural variations in the chromosome 22q11.2 region mediated by nonallelic homologous recombination result in 22q11.2 deletion (del22q11.2) and 22q11.2 duplication (dup22q11.2) syndromes. The majority of del22q11.2 cases have facial and cardiac malformations, immunologic impairments, specific cognitive profile and increased risk for schizophrenia and autism spectrum disorders (ASDs). The phenotype of dup22q11.2 is frequently without physical features but includes the spectrum of neurocognitive abnormalities. Although there is substantial evidence that haploinsufficiency for *TBX1* plays a role in the physical features of del22q11.2, it is not known which gene(s) in the critical 1.5 Mb region are responsible for the observed spectrum of behavioral phenotypes. We identified an individual with a balanced translocation 46,XY,t(1;22)(p36.1;q11.2) and a behavioral phenotype characterized by cognitive impairment, autism, and schizophrenia in the absence of congenital malformations. Using somatic cell hybrids and comparative genomic hybridization (CGH) we mapped the chromosome-22 breakpoint within intron 7 of the *GNB1L* gene. Copy number evaluations and direct DNA sequencing of *GNB1L* in 271 schizophrenia and 513 autism cases revealed dup22q11.2 in two families with autism and private *GNB1L* missense variants in conserved residues in three families (*P* = 0.036). The identified missense variants affect residues in the WD40 repeat domains and are predicted to have deleterious effects on the protein. Prior studies provided evidence that *GNB1L* may have a role in schizophrenia. Our findings support involvement of *GNB1L* in ASDs as well. © 2011 Wiley Periodicals, Inc.

## INTRODUCTION

The chromosome 22q11.2 genomic region harbors four low-copy repeats that make it susceptible to nonallelic homologous recombination that is responsible for the 22q11 deletion (del22q11.2) and duplication (dup22q11.2) syndromes [Stankiewicz and Lupski 2002]. Del22q11.2 is the most common genomic disorder, with a frequency of 1 in 4,000 to 1 in 7,000 births [Botto et al., 2003; Oskarsdottir et al., 2004]. This complex disorder has been called velocardiofacial syndrome (VCFS) [MIM 192430] for its associated facial dysmorphism, palatal clefting or insufficiency, and conotruncal heart abnormalities, or DiGeorge sequence (DGS) [MIM 188400] when the immune system and parathyroid function are compromised. Del22q11.2 also has a prominent cognitive and behavioral component [Murphy 2005]. Individuals with del22q11.2 have a lower IQ than expected and a complex psychoeducational profile characterized by language impairment and higher verbal than performance IQ [Moss et al., 1999; Antshel et al., 2008]. Del22q11.2 greatly increases the risk for psychiatric disorders. An autism spectrum disorder (ASD) is diagnosed in as many as 50% and schizophrenia or related psychosis develops in approximately one-third of individuals with del22q11.2 [Murphy et al., 1999; Fine et al., 2005; Vorstman et al., 2006; Antshel et al., 2007; Niklasson et al., 2009]. Conversely, the microdeletion has been found in approximately 1–3% of adults with schizophrenia and with much higher frequencies in the subsets with childhood onset, making del22q11.2 the most frequent genetic cause of schizophrenia in the general population [Yan et al., 1998; Wiehahn et al., 2004; Horowitz et al., 2005]. Complex neurodevelopmental phenotypes also characterize the newly recognized reciprocal dup22q11.2 syndrome [MIM 608363]. The phenotype of the individuals with dup22q11.2 is exceedingly diverse—some individuals have no apparent abnormalities, but others manifest phenotypes ranging from congenital malformations reminiscent of del22q11.2 to behavioral disorders including intellectual disability, ASD, and learning disorders [Mukaddes and Herguner, 2007; Ramelli et al., 2008; Lo-Castro et al., 2009], further supporting the importance of the 22q11.2 region in cognition and behavior.

Most VCFS/DGS cases have a deletion spanning 3 Mb or, less frequently, a nested deletion of 1.5 Mb, mediated by homologous recombination between different blocks of low-copy repeats. As the range of clinical features in groups with different size deletions is indistinguishable, the boundaries of the 1.5 Mb deletion define a critical del22q11.2 region (NCBI36/hg18 17.2-18.7 Mb) that contains 31 genes [Funke et al., 1999]. Rare patients with unique deletions, translocations or mutations in the region offer an opportunity to dissect the genotypic and phenotypic components of the syndrome. Studies of such patients and of mouse models provide strong evidence that mutations in one of these genes, T-Box1 (*TBX1* [MIM 602054]), can cause the structural craniofacial, cardiovascular, thymic and pharyngeal anomalies, but evidence for the role of TBX1 in the psychiatric/behavioral problems is conflicting [Gong et al., 2000; Yagi et al., 2003; Liao et al., 2004; Torres-Juan et al., 2007; Zweier et al., 2007].

Other genes in the critical del22q11.2 region have also been considered as candidates for the neurobehavioral phenotypes, based on their functions, expression patterns in humans and presence of behavioral abnormalities in mouse models [Gogos et al., 1998; Gothelf et al., 2005; Paterlini et al., 2005]. Recently, significant evidence for association between schizophrenia and Guanine Nucleotide-Binding Protein, Beta-1-Like (*GNB1L* [MIM 610778]) was reported in a case-control association study [Williams et al., 2008]. This observation, combined with the findings of reduced expression of *GNB1L* in postmortem brains of schizophrenics [Ishiguro et al., 2010] and the effect of heterozygous deletion of *Gnbl1* on prepulse inhibition, a schizophrenia endophenotype, in a mouse model [Paylor et al., 2006], suggest that *GNB1L* is associated with the schizophrenia phenotype observed in del22q11.2. Herein we report an individual with a history of autism and schizophrenia who was found to have a balanced translocation involving the 22q11.2 region. We mapped the chromosome 22 breakpoint to the *GNB1L* gene. We also detected additional rare and possibly damaging *GNB1L* sequence variants in subjects with ASD. These findings support the involvement of *GNB1L* in autism.

## MATERIALS AND METHODS

### Proband and Family

The proband and his family members provided informed consent and blood samples under a protocol approved by the University of Washington Institutional Review Board.

### ASD Cases

Cases were identified from families with two or more children with ASD. The ASD cohort is comprised of 513 unrelated individuals, most of whom have participated in our previous genetic studies [Schellenberg et al., 2006; Chapman et al., 2011; Korvatska et al., 2011]. More than 80% of cases are self-identified as White, approximately 10% are of mixed race, and less than 10% are US minorities. The University of Washington and University of Pittsburgh Institutional Review Boards approved the relevant study, and informed consent was obtained from all participants and/or their parents. Children with a reported ASD were assessed using the Autism Diagnostic Interview-Revised (ADI-R) [Lord et al., 1994] and Autism Diagnostic Observation Schedule-Generic (ADOS-G) [Lord et al., 2000] by trained clinicians, and assigned a DSM-IV [APA, 1994] diagnosis. Individuals designated as affected met DSM-IV criteria for an ASD based on ADI-R, ADOS-G, and expert clinical judgment.

### Schizophrenia Cases

The 271 cases were participants in either the 7-site Consortium on the Genetics of Schizophrenia (COGS) or the Veterans Affairs Cooperative Studies Program # 166, Genetic Linkage of Schizophrenia study, and provided informed consent under protocols approved by the Institutional Review Boards at the relevant sites. One hundred fifty five cases were European-Americans and 116 were African-American. For the current project, all cases met DSM-IV criteria for schizophrenia using the Diagnostic Interview for Genetic Studies and related instruments, as described in previous publications [Tsuang et al., 2000; Calkins et al., 2007].

### Control Subjects

Unrelated participants in other studies who had provided informed consent for sharing DNA or genetic information for use in research of other genetic disorders served as controls. These individuals were not screened for autism or schizophrenia. Control subjects were self-identified as European-American (n = 630) or African-American (n = 414).

### Cytogenetic Evaluations

The initial karyotype of the proband with a translocation was analyzed at an outside clinical laboratory. Karyotype and fluorescence in situ hybridization (FISH) analyses of his relatives were performed in the clinical cytogenetics laboratory of the University of Washington.

### Somatic Cell Hybrid Construction

Somatic cell hybrids were constructed using Conversion Technology at the Mayo Clinic Cytogenetics Laboratory [Highsmith et al., 2007]. An EBV transformed lymphoblastoid cell line from the proband was electrofused with E2 cells and 215 mouse/human hybrid colonies were obtained. Twenty-one colonies were expanded and evaluated by FISH for the presence of chromosomes 1 and 22 with whole chromosome paint probes. Separate somatic cell hybrid lines that retained only the derivative chromosome 22 (der22) or the derivative chromosome 1 (der1) and not their normal counterparts were isolated and expanded for DNA extraction.

### Fine Mapping of the Translocation Breakpoints

#### Comparative genomic hybridization (CGH)

DNAs from the separate hybrid cell lines containing either der22 or der1 were sent to NimbleGen Systems (Madison, WI) where they were differentially labeled with either Cy3-dCTP or Cy5-dCTP and hybridized to chromosome-specific oligonucleotide arrays (NimbleGen HG18 Chr1 and HG18 Chr22 designs) according to manufacturer's protocols. Each NimbleGen chromosome specific array contains 385,000 probes. The median probe spacing is 531 base pairs for chromosome 1 and 65 base pairs for chromosome 22. The hybridizations localized the breakpoints at sufficiently high resolution to enable PCR based breakpoint cloning.

#### Capillary sequencing

PCR primers for regions that flanked the breakpoints were designed and tested on normal chromosomes 1 and 22 (Table S I, supplemental material). To establish the exact base-pair positions of the chromosomal breaks on the derivative chromosomes, PCR was then performed using primers that worked during the control runs. For this purpose, the forward primers of chromosome 1 were combined with the forward primers of chromosome 22 to span the breakpoint on der22 and the reverse primers of chromosome 1 were combined with the reverse primers of chromosome 22 to span the breakpoint on the der1. The PCR products were sequenced on an ABI 3130XL DNA analyzer. The PCR and sequencing conditions were the same as described in “Mutation screening” section.

### Detection of Copy Number Variation in *GNB1L* by TaqMan® Copy Number Assay

To detect deletions/duplications of *GNB1L*, we conducted TaqMan® based real-time quantitative copy number tests according to the manufacturer's protocol (Applied Biosystems, Carlsbad, CA). The TaqMan® copy number assay is a duplex reaction with a FAM™-assay targeting the gene of interest and a VIC®-assay targeting the reference gene (Table S II, supplementary material). The copy number is determined by relative quantification using a reference sample known to have two copies of the gene of interest. Each DNA sample was analyzed in triplicate on an Applied Biosystems 7500 Real-Time PCR System. For assay quality, positive control DNA from an individual with del22q11.2 was obtained from the Coriell cell repository (Coriell GM07939). Calculation of gene copy number for each sample was done using the Copy Caller Software V1.0 (Applied Biosystems).

### Confirmation of *GNB1L* Duplication by CGH

Array-CGH experiments were performed as described previously (73), with some modification. Briefly, 250 ng of each of patient and sex matched reference DNA was denatured at 98°C with Cy3- or Cy5-labeled random monomer (TriLink Biotechnologies, San Diego, CA) in 62.5 mM Tris-HCl, pH 7.5, 6.25 mM MgCl2, and 0.0875% b-mercaptoethanol. The denatured sample was chilled on ice, and then incubated with 100 units (exo-) Klenow fragment (NEB, Ipswich, MA) and dNTP mix (6 mM each; Invitrogen, Carlsbad, CA) in Tris, EDTA buffer, for 2 hr at 37°C. Reactions were terminated by addition of 0.5 M EDTA (pH 8.0); the end products were precipitated with isopropanol and resuspended in water. The Cy-labeled test sample and reference samples were then combined (10 µg each) and hybridized to a custom-designed (Girirajan and Eichler unpublished) 400 K oligonucleotide array (Agilent Technologies, Santa Clara, CA) at 60°C for 40 hr. The arrays were washed in commercially available solutions and scanned. The resulting TIFF images were analyzed with Feature Extraction software and output from this software was imported into CGH Analytics software for final analysis. The data were analyzed with the Genomic Workbench using an ADM-2 algorithm with a 0.25 cut-off threshold. The wash buffers, scanner, and all software are available from Agilent Technologies.

### Mutation Screening by Capillary Sequencing

For ASD and schizophrenia cases and 130 controls, DNA was extracted from lymphocytes and lymphoblastoid cell lines. Primers flanking coding exons were designed with Primer3 (Table S III, supplemental material). Genomic DNA was PCR amplified in a MJ Research DNA Engine Tetrad 2. The 15 µl final volume contained 40 ng DNA, 0.3 µM each primer, 0.5 U Qiagen HotStarTaq DNA Polymerase, 1.5 mM MgCl2, and 200 µM each dNTP. The conditions consisted of an initial incubation of 95°C for15 min, followed by 30 cycles at 94°C for 45 sec, 60°C for 45 sec, and 72°C for 60 sec. The final incubation step was at 72°C for 7 min. Aliquots of 5 µl of PCR amplified DNA fragments were prepared for sequencing with Exonuclease I/Shrimp Alkaline Phosphatase digestion using 2 µl of USB Corporation ExoSAP-IT for 30 min at 37°C followed by enzyme deactivation at 80°C for 15 min. A 2 µl aliquot of the ExoSAP-IT treated PCR product was cycle sequenced with Applied Biosystems BigDye Terminator v3.1 on a MJ Research DNA Engine Tetrad 2. The conditions were, 10 µl final volume, 1.5 µl Big Dye, 4 pmol primer, and 2 µl of 5 M Betaine, initial incubation of 96°C for 2 min followed by 25 cycles at 95°C for 15 sec, 50°C for 10 sec, and 60°C for 4 min. The dye labeled sequencing reaction products were capillary electrophoresed on a Applied Biosystems 3130XL Genetic Analyzer with a 36 cM array, 3130 POP-7 polymer, 3130XL Data Collection v3.0 and Sequencing Analysis Software v5.3.1. Further analysis was performed with SeqScape 2.1 or DNASTAR Lasergene 8.1.

### Mutation Screening by Next-Generation Sequencing

Exome sequence data were produced by the University of Washington Genome Sciences Genomic Resource Center using the same methods for exome capture, massively parallel sequencing, read mapping, and annotation as previously described [Norton et al., 2011]. Sequences were analyzed across the *GNB1L* gene for coding exons 3–8 for 500 self-identified European-Americans and 414 African-Americans.

### Mutational Burden Analysis

In concordance with work on Mendelian disorders where rare variants of large effects are causal, our assumption was that potentially pathogenic *GNB1L* variants would be rare changes that are evolutionary conserved and predicted to have deleterious effects on protein function. To evaluate evolutionary conservation of discovered variants we used PhastCons [Siepel et al., 2005] and GERP [Goode et al., 2010], and to evaluate putative effects of amino acid change on protein function we used Polyphen [Ramensky et al., 2002], pMUT [Ferrer-Costa et al., 2005], and SIFT [Ng and Henikoff, 2003]. To deem a variant potentially pathogenic, we required it to be determined as evolutionarily conserved with at least one of the two methods, and considered damaging with at least two of the three methods.

## RESULTS

### Proband and Family

The proband was seen in the Genetic Medicine Clinic at the University of Washington for deteriorating cognitive and social functioning. He had a history of autism as a child, schizophrenia as a teenager and a persistent, variably severe problem with speech production. The perinatal period was marked by emergency cesarean section at 29 weeks gestation, birth weight of 1,304 g, and 2 months hospitalization. Despite these complications, he met developmental milestones, walked at approximately 1 year, and spoke single and then combined words between 1 and 2 years. However, in early childhood he had a tendency to memorize phrases, paced in circles, and appeared to “imagine things.” In kindergarten he was found to have receptive and expressive language delays that qualified him for special education services and school records indicate that in the first grade he was evaluated at the Child Development and Rehabilitation Center at Oregon Health and Science University and diagnosed with autism. Cognitive testing was performed on several occasions. Full scale Wechsler Intelligence Scale for Children III (WISC-III) [Wechsler, 1992] IQ scores of 76 and 87 were obtained at ages 6.2 and 7.6 years, respectively. At 16.5 years performance tests from the Wechsler Individual Achievement Test II (WIAT-II) [Wechsler, 2002] demonstrated a wide range of scores, from 111 (77th percentile) for phonological decoding to 40 (<0.1 percentile) for math reasoning.

In the public school system he received special education and speech services. The deterioration of expressive language was noted, and he never spoke more than one or two words at a time. In the 8th grade, he began acting confused, his behavior became more erratic with episodes of anger and violence, and he reported hearing voices and “beeping noises.” At age 14 he was hospitalized with visual, olfactory hallucinations, and dysregulated behavior and a diagnosis of schizophrenia was made. Although his behavior stabilized on a combination of antipsychotic medications, he continued to be intermittently agitated and disorganized and to complain of auditory hallucinations. In later teen years, he became more anxious and was started on a selective serotonin reuptake inhibitor. There is no history of seizures and no seizure activity was detected on multiple EEGs. A recent brain MRI was normal.

On examination by a medical geneticist and a child and adolescent psychiatrist at the University of Washington at age 18 no physical features of VCFS were detected. The neurologic exam was normal. The patient was casually dressed and cooperative but avoided eye contact, except when he was directly addressed. He sat somewhat restlessly in the chair, with stereotypic movements characterized by rubbing his hands back and forth over his knees. At times, it appeared that he was gesticulating with his mouth as if he were talking to someone. It appeared that he is struggling with speech production. He would respond verbally to some questions, but only after a long delay and with only one or two words, making mental status examination difficult. It was notable that there was no delay in his nonverbal responses. The clinical diagnosis by the psychiatrist was pervasive developmental disorder not otherwise specified and psychosis not otherwise specified. A structured psychiatric evaluation was not performed and available records were insufficient for establishment of a structured diagnosis.

### Cytogenetic Evaluations

**FIG. 1:**
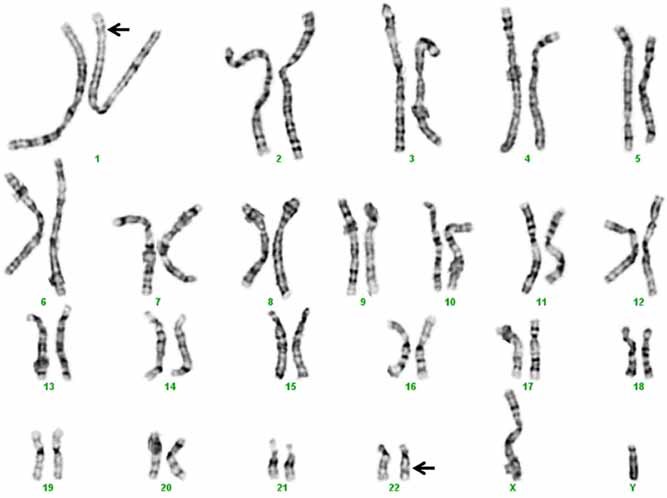
Karyotype showing the balanced translocation t(1;22)(p36.1;q11.2) in the proband and other members of his family. Arrows indicate derivative chromosomes 1 and 22.

Karyotype analysis revealed a balanced translocation, t(1;22)(p36.1;q11.2) (Fig. 1). FISH was performed in the University of Washington clinical laboratory with two probes for chromosome 22q. Signals for the 22q11.2 VCFS probe were observed on both the normal and derivative chromosomes 22, while signals from the distal 22q13.3 ARSA probe were observed on the normal chromosome 22 and derivative chromosome 1 (Fig. S1, supplementary material). Additional cytogenetic evaluations revealed that the proband's father and several other paternally related family members are balanced translocation carriers, which is consistent with the history of infertility and multiple pregnancy losses. There was no history of developmental disability, autism or psychosis in these or other relatives, but neuropsychiatric testing was not done. A pedigree is not shown because of concerns regarding identifiability.

### Characterization of the Translocation Breakpoints

**FIG. 2:**
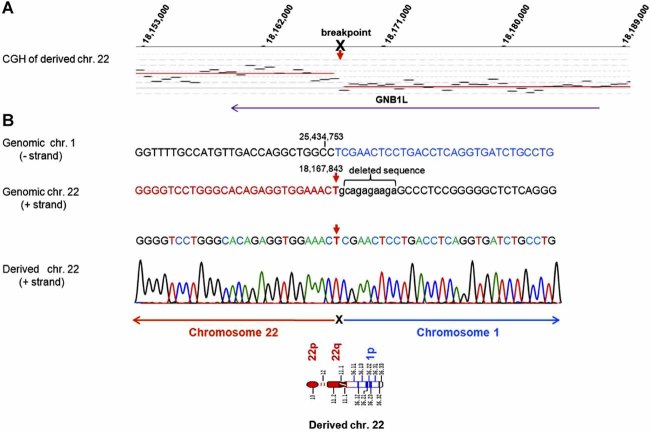
Fine mapping of the chromosome 22 translocation breakpoint. The derivative chromosome 22 was differentially labeled with Cy3-dCTP, derivative chromosome 1 was labeled with Cy5-dCTP and both were hybridized to chromosome 22 specific oligonucleotide array (NimbleGen HG 18 Chr22 design). The hybridization localized the breakpoint at sufficiently high resolution (**A**) to enable PCR based breakpoint cloning (**B**). The chromosome 22 breakpoint is between 18,167, 843-18, 167,854 bp, within intron 7 of the GNB1L gene, and encompasses a 10 bp deletion shown in lower case (tcttctctgc).

CGH with somatic cell hybrids that each contained one derivative chromosome was used to fine map the translocation break points. To precisely determine the translocation location, primers spanning the breakpoint were designed and the region was sequenced. The chromosome 1 breakpoint is at 25,434,753 bp (NCBI36/Hg18). (Fig. S2, supplementary material). The chromosome 22 breakpoint is at 18,167,843-18,167,854, encompassing a 10 bp deletion (tcttctctgc) (Fig. 2). The chromosome 1 breakpoint lies in a heterochromatic region of ALU repetitive DNA between the *SYF2* and *C1orf63* genes. The chromosome 22q11.21 breakpoint lies within intron 7 of the *GNB1L* gene, a region without repetitive DNA. No homology was found between the two breakpoints. *GNB1L* has 8 exons spanning 66.5 kb of genomic DNA encoding 327 amino acids (NP_443730). The fusion predicts substitution of 16 amino acids from the noncoding ALU sequence of chromosome 1 in place of the terminal 83 amino acids of GNB1L. No other abnormalities of sequence dosage for chromosomes 1 or 22 were detected by whole genome CGH (data not shown).

### Evaluation of *GNB1L* for CNVs and Mutational Burden Analysis

Using Quantitative TaqMan ® Copy Number Assays we detected *GNB1L* duplications in two subjects with ASD. By array CGH we determined that both are the typical 3 Mb dup22q11.2.

**FIG. 3:**
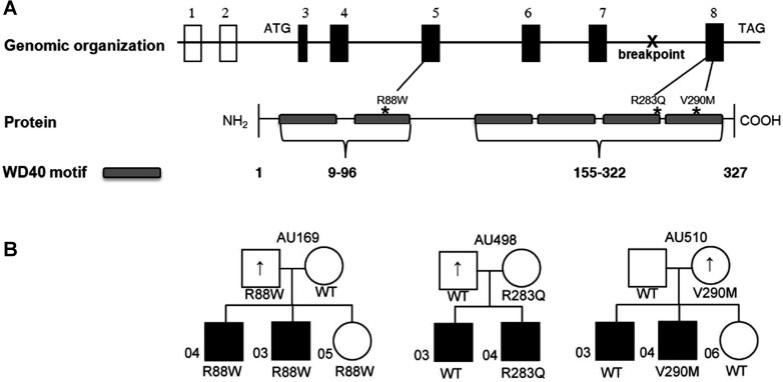
Schematic of *GNB1L* and corresponding protein and transmission of *GNB1L* variants in families with autism. **A**: The chromosome 22 translocation breakpoint lies in intron 7. The locations of the three unique variants found in ASD in the WD40 motifs are indicated. **B**: Affected individuals in all families have a diagnosis of autism or ASD, indicated by black fill. Up-arrow indicates an elevated BPASS social motivation and range of interest/flexibility scores; scores have a direct correlation with symptoms of ASD such that high scores are abnormal.

We sequenced the 6 coding exons and splice junctions of *GNB1L* in genomic DNA of 271 subjects with schizophrenia and 513 subjects with ASD. In this sample we identified five missense variants we define as rare as they are not listed in the NCBI dbSNP Build 132 (R57Q, C70R, R88W, R283Q, and V290M). Two of these variants R57Q and C70R, were detected in multiple controls of European-American and African-American background and are likely to be polymorphisms. The three private variants found in ASD subjects, R88W, R283Q, and V290M, affect residues in the WD40 domains and meet our criteria for missense variants that are highly conserved across multiple species as determined by PhastCons and GERP, and predicted to have pathogenic effects on the protein structure by at least two of three software algorithms (SIFT, PolyPhen, and pMUT). No rare variants were detected in schizophrenia sample. For a control sample we have evaluated coding sequence of 630 controls of European-American and 414 controls of African-American ancestry. In this combined sample of 1,044 controls, we have identified four variants that were not present in dbSNP Build 132, none of which have met our criteria for deleteriousness based on conservation score and effects on protein function (Fisher Exact, two sided *P* = 0.036) (Table I). Figure 3A shows the genomic organization of *GNB1L* and the protein location of unique variants identified in ASD cases.

**TABLE I:** Summary of Rare Missense Variants Detected in ASD Cases and Unscreened Controls

				Prediction of Functional Effect			Evolutionary conservation	
					
Nucleotide change	Protein residue	hg18 location	Number of variants	Polyphen (40, 41)	pMUT (42)	SIFT (39)	Phast cons^a^	GERP^b^
ASD cases (n = 513)								
c.262G/A	p.R88W	18,179,963	1	Damaging	Damaging	Damaging	1	4.61
c.848C/T	p.R283Q	18,156,368	1	Benign	Damaging	Damaging	1	0.938
c.868C/T	p.V290M	18,156,348	1	Damaging	Benign	Damaging	0.997	4.97
Controls (n = 1,044)								
c.17G/A	p.P6L	18,188,862	1	Benign	Benign	Benign	0	−4.38
c.313C/T	p.V108M	18,179,903	1	Damaging	Benign	Benign	0.068	0.158
c.692G/A	p.A231V	18,169,564	1	Benign	Benign	Benign	0.988	2.91
c.913G/A	p.A305T	18,156,303	1	Benign	Benign	Benign	0	−9.54

a PhastCons scores range from 0 to 1, where 1 is most conserved.

b GERP conservation scores range from −11.6 to 5.82, where 5.82 is most conserved.

### Transmission in Families

**FIG. 4:**
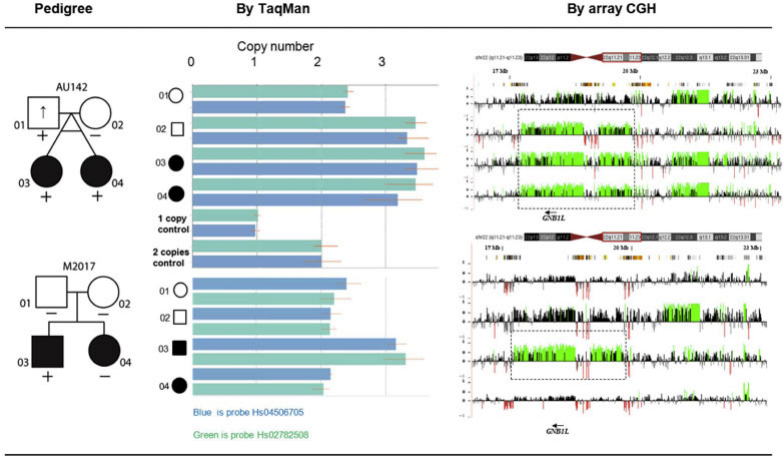
Identification of *GNB1L* duplication and transmission patterns in two families with autism. Affected individuals in the pedigrees have a diagnosis of autism or ASD, indicated by black fill. Up-arrow indicates elevated BPASS social motivation and range of interest/flexibility scores. (+) indicates 22q11.2 duplication shown as increased copy number by TaqMan and CGH.

The duplications identified in the probands were genotyped in all available members of both families (Fig. 4). In family AU142, the 3 Mb duplication was transmitted from the father to both affected monozygotic twin daughters, one of whom had autism and the other had a diagnosis of ASD. Both had low IQ scores (full-scale IQ 49). The father showed decreased social motivation and limited range of interests/flexibility as assessed by the Broader Phenotype Autism Symptom Scale (BPASS) [Dawson et al., 2007]. In family M2017 the duplication is a de novo event in one of the two affected offspring with a diagnosis of autism.

All three private missense variants were transmitted from an unaffected parent (Fig. 3B). In family AU169, both affected sons with autism and the unaffected daughter inherited the R88W variant; the carrier father had elevated BPASS scores, indicating presence of the broader autism phenotype. In families AU498 and AU510, a brother with ASD did not carry the respective R283Q or V290M variant. The carrier mother in family AU510 showed subclinical traits of the autism phenotype as reflected by an elevated BPASS score, as did the noncarrier father in family AU498.

## DISCUSSION

There is increasing evidence that autism and schizophrenia are highly heterogeneous disorders on both phenotypic and genotypic levels [McClellan et al., 2007; Abrahams and Geschwind, 2008; Bassett et al., 2010; State, 2010]. Recent molecular studies have identified multiple risk factors for both disorders. For autism there is evidence for a pathogenic effect of missense mutations in a number of genes, including *NLGN3* (MIM 300336) and *NLGN4* (MIM 300427) [Jamain et al., 2003], *NRXN1* (MIM 600565) [Kim et al., 2008], *SHANK3* (MIM 606230) [Durand et al., 2007], *SHANK2* (MIM 603290) [Berkel et al., 2010], and *CNTNAP2* (MIM 604569) [Bakkaloglu et al., 2008]. In addition to the 22q11.2 region, recurrent deletions and duplications at 7q11.3, 15q11–q13, 16p11.2, and 17p11.2 have autism as a frequent phenotypic presentation [Cook and Scherer 2008; Stankiewicz and Lupski, 2010). None of these single gene mutations or CNVs account for more than a minority of autism cases. Similarly, for schizophrenia, the list of candidate genes and CNVs continues to increase [Bassett et al., 2010; Stankiewicz and Lupski, 2010]. To date, the cumulative results point to involvement of many genes and unknown environmental influences on the pathogeneses of autism and schizophrenia and the variability in their phenotypic expression.

Although deletion of the 22q11.2 region is associated with autism and is the most common identified cause of schizophrenia, it is not known which gene or genes confer this risk. The disruption of *GNB1L* by a balanced translocation in the del22q11.2 region in our proband with ASD as a child and schizophrenia as a young adult suggests that this gene might play a role in neurobehavioral phenotypes. Williams et al. 2008 reported significant evidence for a male specific association between schizophrenia and genotypes at the *GNB1L* locus and replicated these findings in two independent samples with a total of 1,408 cases and 2,746 controls. Furthermore, they found a significant allelic association between the variants of *GNB1L* and psychosis in males with del22q11.2. By direct sequencing of the *GNB1L* coding exons, they did not identify any novel variants, but this was a limited re-sequencing study of only 14 subjects. The association of common *GNB1L* alleles and schizophrenia was recently replicated in a Chinese sample [Li et al., 2011]. *GNB1L* (MIM 610778) encodes a 327 amino acid G-protein beta-subunit-like polypeptide that belongs to the WD-motif repeat protein family. GNB1L protein contains six putative WD40 repeats and no other recognizable functional domains [Gong et al., 2000]. WD-motif containing proteins have 4–16 repeating units, all of which are thought to form a circularized beta-propeller structure. Members of the WD-motif family are found in all eukaryotes and are involved in a variety of cellular processes, including cell cycle progression, signal transduction, apoptosis, and gene regulation. WD-repeat proteins have been associated with inherited neurodevelopmental and neurodegenerative diseases, such as lissencephaly-1 (MIM 607432), Parkinson disease type 8 (MIM 607060), Cockayne syndrome type A (MIM 216400), Triple-A syndrome (MIM 231550), microcephaly (MIM 600176), and Joubert Syndrome Type 3 (MIM 608629). In mice, Gnb1l is an essential protein as homozygous loss of function causes embryonic lethality [Paylor et al., 2006]. It is widely expressed in the forebrain, midbrain, and hindbrain structures of the adult mouse, and hemizygous deletion of *Gnb1l* is associated with deficits in prepulse inhibition of the startle response, a schizophrenia endophenotype [Braff et al., 2001].

All of these factors motivated our evaluation of the contribution of *GNB1L* variants to autism and schizophrenia. As large multigenic 22q11.2 CNVs are responsible for del22q11.2 and dup22q11.2 genomic disorders, we first evaluated *GNB1L* for copy number changes using TaqMan assays that could, in principle, detect smaller single gene CNVs. In this fashion we have detected two *GNB1L* duplications in ASD subjects that we confirmed with CGH as 3 Mb multigenic duplications. As is true for the del22q11.2 syndrome, the newly recognized dup22q11.2 syndrome is characterized by cognitive and behavioral disturbances and extensive phenotypic variability [Ensenauer et al., 2003; Yobb et al., 2005; Mukaddes and Herguner, 2007; Ou et al., 2008; Yu et al., 2008; Lo-Castro et al., 2009]. In one of our families the dup22q11.2 was transmitted from a parent who had elevated BPASS scores for social motivation and range of interest/flexibility. The BPASS was developed for use with child and adult relatives as well as affected children to assess social motivation, social expressiveness, conversational skills, and flexibility, domains that are abnormal in ASD [Dawson et al., 2007]. The BPASS scores were designed to span the range from clinical impairment to typical behavior and have a direct correlation with symptoms of ASD such that high scores indicate abnormality or impairment in autism related traits. Although the father had normal cognition, his monozygotic twin daughters share low IQ scores but differ with respect to severity of behavioral impairment. In the second family one of the two autistic siblings has a de novo duplication, suggesting that in this family additional risk factors are present. We are not aware of any publication that has found an association of dup22q11.2 with schizophrenia and we did not detect either deletion or duplication of 22q11 in our relatively small sample of 271 schizophrenic subjects. It is interesting to note that the del22q11.2 is associated with schizophrenia and autism and dup22q11.2 has been described only in autism, suggesting shared genetic influences modulated by gene dosage.

Our second assumption was that rare coding sequence changes in *GNB1L* might be responsible for the phenotype in a subset of ASD or schizophrenia cases. In 513 subjects with ASD we found three rare missense *GNB1L* variants that affect highly conserved nucleotides and are predicted to be deleterious. We have found no such variants in a smaller sample of 271 schizophrenia cases. For mutational burden analysis we defined potentially pathogenic variants as not present in dbSNP, conserved and as having deleterious effects on protein function as determined by two out of three bioinformatic functional prediction tools. Our assumption is that variants defined in such a way that includes nucleotide-sequence conservation and protein based prediction of functional effects are likely to be deleterious regardless of the ethnic background of subjects. The three ASD variants met those criteria and no variants meeting these criteria were detected in 1,044 controls (*P* = 0.036). The unique *GNB1L* variants in our families with autism were all inherited. As with dup22q11.2, the variants did not co-segregate with ASD diagnoses, suggesting the presence of additional risk factors. In autism males outnumber females by approximately fourfold, possibly a consequence of a lower threshold for expression of the ASD phenotype in males. This hypothesis is consistent with the transmission patterns we have observed. In the three autism families with missense mutations four of the five males who carry a variant have autism or ASD and the fifth has an elevated BPASS score. In contrast, only one of three female carriers has an elevated BPASS score. Our findings are consistent with the hypothesis that *GNB1L* mutations are risk factors in a multigenic threshold model for autism. Given the decreased penetrance and marked variability in expression for the behavioral phenotypes observed with deletion and duplication of the 22q11.2 region, it is likely that any causal gene in the region would have decreased penetrance as well. Decreased penetrance and variable phenotypic expression have been observed for other genes in which rare variants are reported to be associated with autism [Abrahams and Geschwind, 2008; State, 2010] such as *SHANK3* [Moessner et al., 2007], *NRXN1* [Kim et al., 2008], and *CNTNAP2* [Bakkaloglu et al., 2008].

Our study has several weaknesses. Although the 46,XY,t(1;22)(p36.1;q11.2) translocation disrupts the *GNB1L* gene, it is possible that the resulting phenotype is due to position effects that alter expression of genes in the vicinity including *TBX1* and *COMT* which are also strong candidates for behavioral phenotypes. In our screening of *GNB1L* for potentially damaging sequence variants we found an association of rare potentially damaging nonsynonimous variants and ASD. This conclusion is based on the assumption that currently available bioinformatic tools can accurately distinguish variations that will change the protein function and ultimately result in a behavioral phenotype. An additional weakness is that our cases and controls contain several ethnic and racial groups and their ethnic classification is based on self-identification. It is possible that the variants we have identified as potentially deleterious are polymorphisms in one of the ethnic subpopulations. Our findings should be replicated, preferably in a larger sample of cases, as it is likely that the contribution of rare variants in any one gene is responsible for a very small proportion of the genetic burden for complex disorder such as autism.

If our finding that rare missense *GNB1L* variants contribute to ASD in a subset of cases is replicated, studies would be warranted to determine the effects of such mutations on the GNB1L protein whose function is not well understood. As *GNB1L* lies in the critical del22q11.2 region it is a priori a candidate gene for neurodevelopmental disorders that occur in the associated CNV syndromes. Coupled with observations of other investigators for its involvement in schizophrenia, our detection of a translocation disrupting *GNB1L* and potentially damaging, extremely rare *GNB1L* variants in subjects with autism provides evidence that this gene may have a role in the pathogenesis of autism and expands the number of phenotypes associated with *GNB1L*.
