# Supplementary figures and images for: Evidence for Involvement of GNB1L in Autism
Source: Am J Med Genet B Neuropsychiatr Genet. 2011 Nov 16;159B(1):61–71. doi: 10.1002/ajmg.b.32002 (PMC3270696; doi:10.1002/ajmg.b.32002)

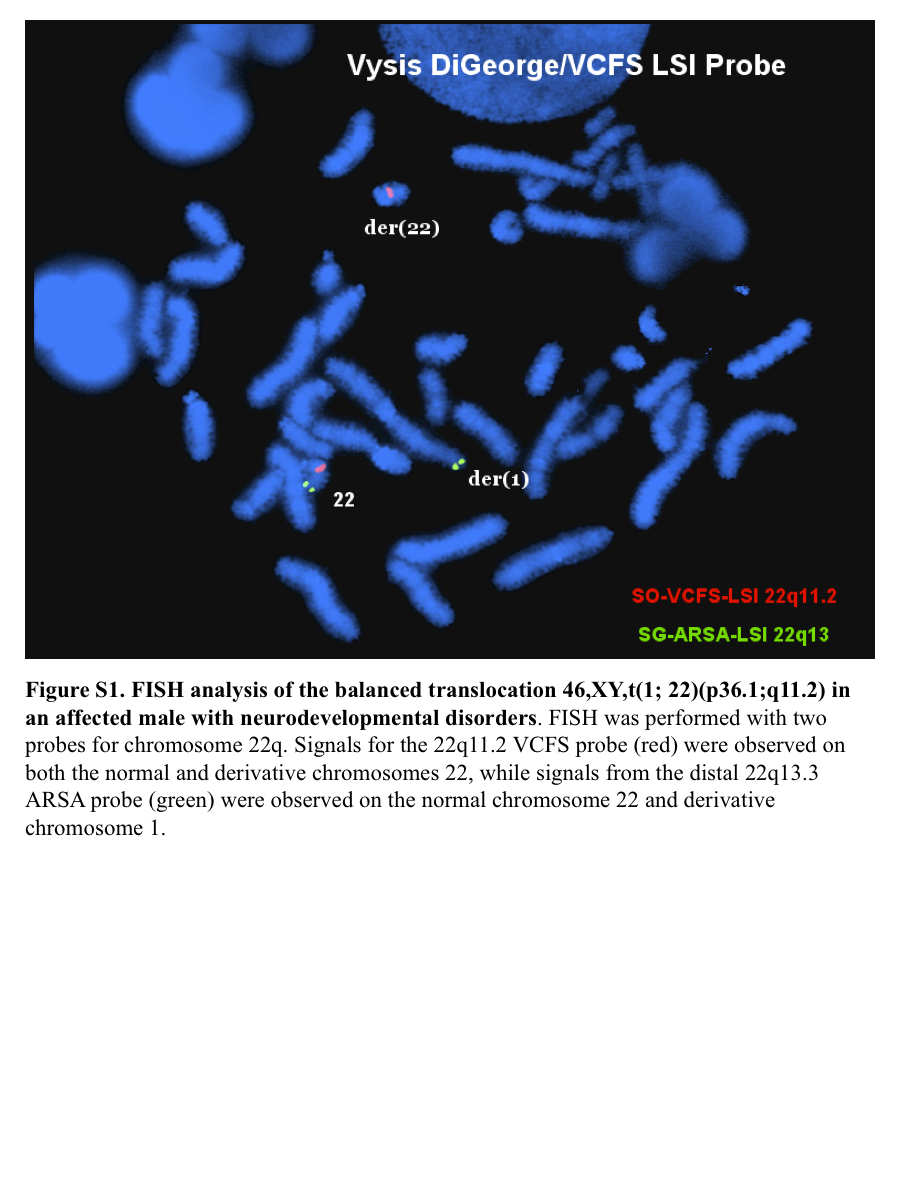

Supplement: Supplementary file 1 [file ajmg159B-0061-SD1.tiff]

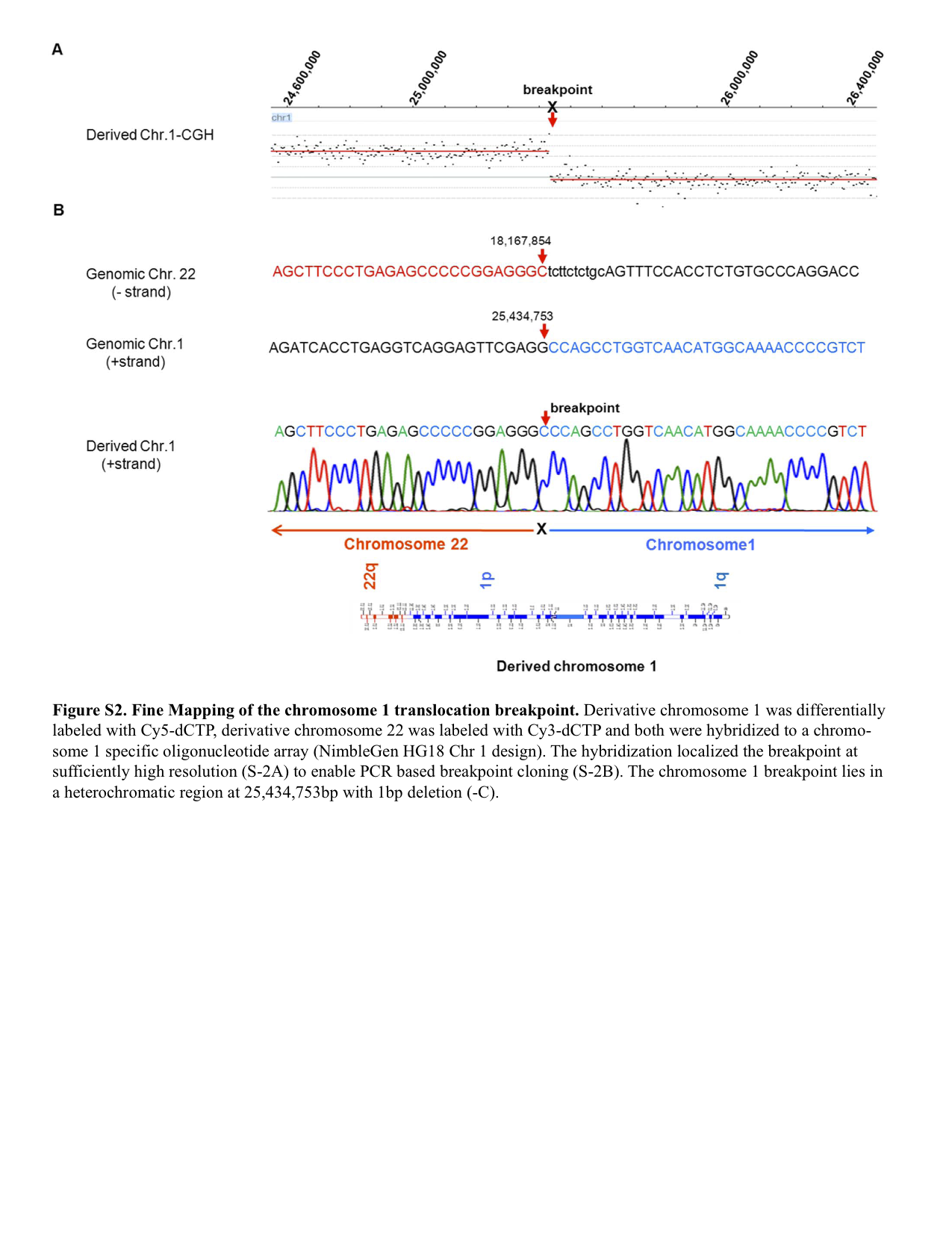

Supplement: Supplementary file 2 [file ajmg159B-0061-SD2.tiff]
