# Supplementary material for: Evidence for Involvement of GNB1L in Autism
Source: Am J Med Genet B Neuropsychiatr Genet. 2011 Nov 16;159B(1):61–71. doi: 10.1002/ajmg.b.32002 (PMC3270696; doi:10.1002/ajmg.b.32002)
Supplement: Supplementary file 3 [file ajmg159B-0061-SD3.doc]

**Table S I: Primers used to map the breakpoints**

| Primer name | Sequence | NCBI 36 hg18 | Chromosome | Size (bp) |
| --- | --- | --- | --- | --- |
| Derived Chr.1_BP1_F5 | 5´-TAAGGCATTTACTCTTTGACTCAGC-3´ | 25434125 -25434149 | 1 | 758 |
| Derived Chr.1_BP22_F3 | 5´-GAACATGCCCCATAAATTACAGTC-3´ | 18167712 -18167735 | 22 |  |
| Derived Chr.22_BP1_R9 | 5´-ATACTGTTGACTATTCTTAGTTTTTCCA-3´ | 25435023 -25435050 | 1 | 1163 |
| Derived Chr.22_BP22_R8 | 5´-GGTCAGATTGCCCAGTCTAGTG-3´ | 18168700 -18168721 | 22 |  |

**Table S II: TaqMan®copy number assay probes**

| Assay ID | Context Sequence | Location on NCBI 36 hg18 |
| --- | --- | --- |
| Hs04506705_cn | ACCCCCCACCTCCACCGCCCAAATG | 18164692 |
| Hs02782508_cn | GCGGCTGCACACCTTCTGCTCAGAG | 18169660 |

**Table S III: Primers for GNB1L mutation screening**

| Primer Name | Sequence | Size (bp) | Tm |
| --- | --- | --- | --- |
| Exs1+2_GNB1L_F | 5'-GAGAGCCTGCGAGGTAGC-3' | 627 | 62°C |
| Exs1+2_GNB1L_R | 5'-GCAGGAAATACGAGCTCACAA-3' |  |  |
| Exon_3-GNB1L-3F | 5'-GAACTGGTGCCTCTGTAGCC-3' | 409 | 58°C |
| Exon_3_GNB1L-3R | 5'-GGGCTCCTTGCTAGCTCAC-3' |  |  |
| Exon_4_GNB1L-4F | 5'-AGAGCTGTTGGCTTTTCAGC-3' | 467 | 58°C |
| Exon_4_GNB1L-4R | 5'-AGTGGAGATGGTGGAGATGC -3' |  |  |
| Exon_5_GNB1L-5F | 5'-TTGATCCACTGCATGTCCTG-3' | 485 | 58°C |
| Exon_5_GNB1L-5R | 5'-ATGGAGTCCTGTGGGTAGGC-3' |  |  |
| Exon_6_GNB1L-6F | 5'-ACAGAAACTCACAGCAGCCC-3' | 298 | 58°C |
| Exon_6_GNB1L-6R | 5'-AGTGGCTCGACGATAAATCAG-3' |  |  |
| Exon_7_GNB1L-7F | 5'-ACGTGGAAGACATGGGAAAC-3' | 443 | 58°C |
| Exon_7_GNB1L-7R | 5'-GGGATCTGCAACAACTCCTC-3' |  |  |
| Exon_8_GNB1L-8F | 5'-TCCCTGTCTCTGCTGCTCTC-3' | 635 | 58°C |
| Exon_8_GNB1L-8R | 5'-CATGACTTGCTGGTCCTCAG-3' |  |  |
